# Boosting the Synthesis of Pharmaceutically Active Abietane Diterpenes in *S. sclarea* Hairy Roots by Engineering the *GGPPS* and *CPPS* Genes

**DOI:** 10.3389/fpls.2020.00924

**Published:** 2020-06-18

**Authors:** Maria Carmela Vaccaro, Mariaevelina Alfieri, Nunziatina De Tommasi, Tessa Moses, Alain Goossens, Antonietta Leone

**Affiliations:** ^1^Department of Pharmacy, University of Salerno, Fisciano, Italy; ^2^Department of Plant Biotechnology and Bioinformatics, Ghent University, Ghent, Belgium; ^3^VIB Center for Plant Systems Biology, Ghent, Belgium

**Keywords:** metabolic engineering, plant bioactive diterpenes, gene co-expression, silencing, MEP-pathway

## Abstract

Abietane diterpenoids (ADs), synthesized in the roots of different *Salvia* species, such as aethiopinone, 1-oxoaethiopinone, salvipisone, and ferruginol, have a variety of known biological activities. We have shown that aethiopinone has promising cytotoxic activity against several human tumor cell lines, including the breast adenocarcinoma MCF7, HeLa, epithelial carcinoma, prostate adenocarcinoma PC3, and human melanoma A375. The low content of these compounds in natural sources, and the limited possibility to synthesize them chemically at low cost, prompted us to optimize the production of abietane diterpenoids by targeting genes of the methylerythritol phosphate (MEP) pathway, from which they are derived. Here, we report our current and ongoing efforts to boost the metabolic flux towards this interesting class of compounds in *Salvia sclarea* hairy roots (HRs). Silencing the gene encoding the *ent*-copalyl-diphosphate synthase gene (ent*CPPS*), acting at the lateral geranylgeranyl pyrophosphate (GGPP) competitive gibberellin route, enhanced the content of aethiopinone and other ADs in *S. sclarea* HRs, indicating indirectly that the GGPP pool is a metabolic constraint to the accumulation of ADs. This was confirmed by overexpressing the *GGPPS* gene *(*geranyl-geranyl diphosphate synthase) which triggered also a significant 8-fold increase of abietane diterpene content above the basal constitutive level, with a major boosting effect on aethiopinone accumulation in *S. sclarea* HRs. A significant accumulation of aethiopinone and other AD compounds was also achieved by overexpressing the CPPS gene (copalyl diphosphate synthase) pointing to this biosynthetic step as another potential metabolic target for optimizing the biosynthesis of this class of compounds. However, by co-expressing of *GGPPS* and *CPPS* genes, albeit significant, the increase of abietane diterpenoids was less effective than that obtained by overexpressing the two genes individually. Taken together, the results presented here add novel and instrumental knowledge to a rational design of a hairy root-based platform to yield reliable amounts of aethiopinone and other ADs for a deeper understanding of their molecular pharmacological targets and potential future commercialization.

## Introduction

Abietane diterpenes (ADs), extracted from a variety of plant sources, display a wide variety of biological activities, including antibacterial, antioxidant, anti-inflammatory, and antifungal activities, amongst others (Çadirci et al., 2012; Wu et al., 2012; Hanson, 2013; Gonzalez, 2015). In particular, the antiproliferative activity of both phenolic-type (carnosic acid and ferruginol) and quinone-type (aethiopinone, salvipisone, and 1-oxoaethiopinone) ADs has been reported in different cancer cell lines (Gonzalez, 2015; Akaberi et al., 2015; Esquivel et al., 2017). Aethiopinone and salvipisone from *S. sclarea* induce caspase-3 mediated apoptosis in a time- and concentration-dependent manner in leukemia cells (Rozalski et al., 2006). We have also demonstrated that aethiopinone is cytotoxic to the solid tumor MCF7 (breast adenocarcinoma), HeLa (epithelial carcinoma), and PC3 (prostate adenocarcinoma) cell lines, while being safe for non-malignant cells (Vaccaro et al., 2014). Interestingly, aethiopinone was also able to induce apoptosis in A375 (human melanoma) cells (Vaccaro et al., 2014), encouraging further investigation of this compound as a promising anti-tumor compound, particularly against resistant melanoma cells (Kalal et al., 2017). However, aethiopinone and other bioactive ADs in general accumulate in *S. sclarea* roots at very low level (< 0.5% DW), preventing further studies to better understand their molecular targets and potential pharmaceutical use. To achieve suitable commercial supplies of high-value plant secondary metabolites, several strategies have been designed and developed for their engineered bio-production in suitable plant and/or heterologous systems (Lu et al., 2016; Vavitsas et al., 2018). Plant cell and hairy root (HR) cultures have also received increasing attention as a valuable option to large-scale production systems for high-value plant secondary metabolites, because they offer the advantage of extraction under controlled conditions coupled to a strict quality control (Tian, 2015; Hidalgo et al., 2018).

**Figure 1:**
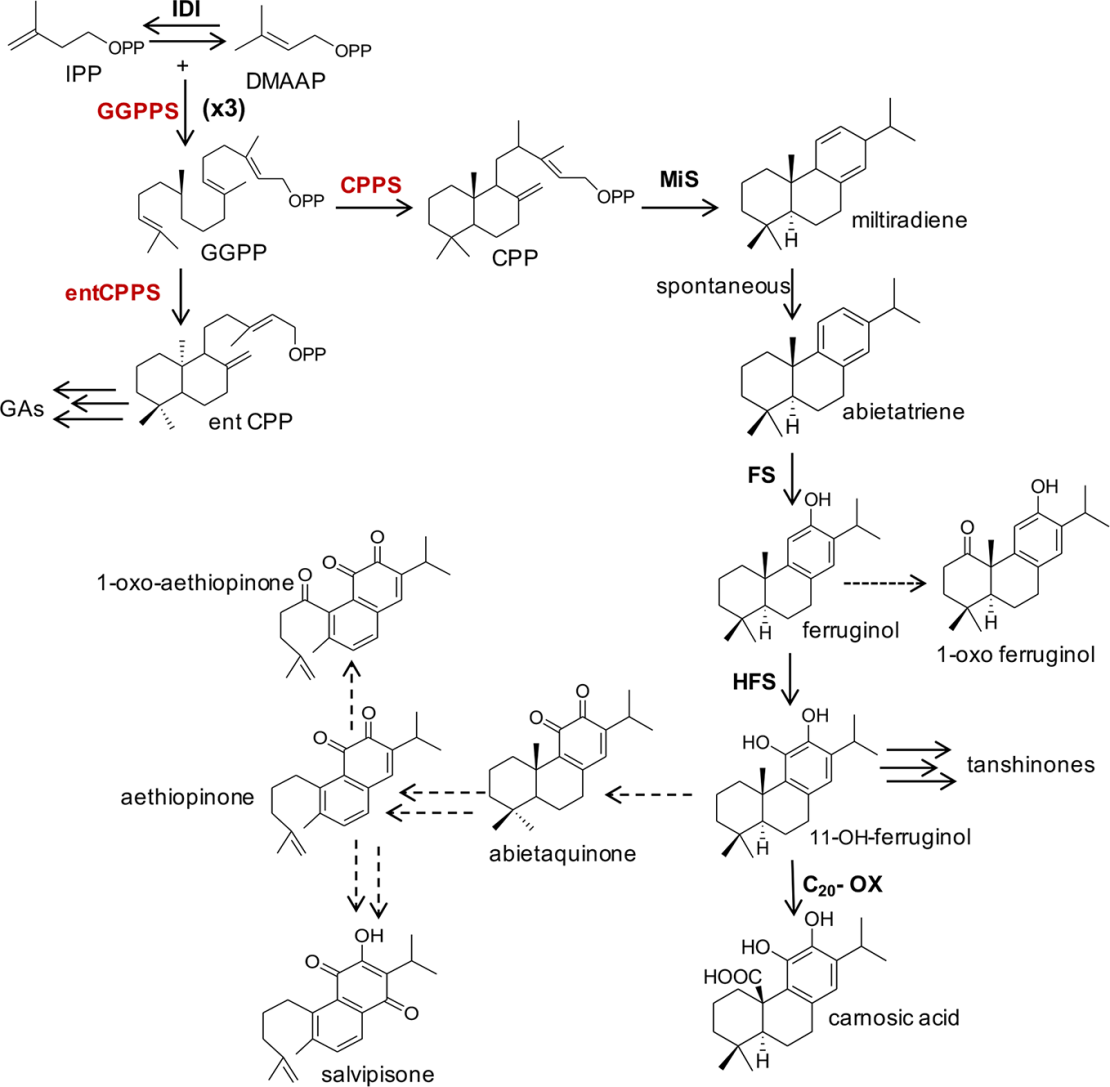
A simplified schematic representation of plastidial MEP-pathway to abietane diterpenes and other isoprenoids derived from GGPP in Lamiaceae species. The main enzymatic steps (bold letters), intermediates and final products are indicated. The terpene synthases CPPS and MiS cyclize GGPP to miltiradiene, which spontaneously oxidizes to abietatriene. Cytochrome P450 monooxygenases (FS and HFS), oxidize abietatriene to ferruginol and 11-hydroxyferruginol, through two successive oxidations. A further oxidation of 11-hydroxyferruginol position at the C_20_, by a C_20_-oxidase, leads to carnosic acid. A possible biosynthetic route of aethiopinone, 1-oxo-aethiopinone, and salvipisone is also shown. Dashed lines indicated hypothesized chemical conversions and unknown enzymatic steps. The genes targeted by metabolic engineering in this study are indicated in red. MEP, 2-C-methyl-D-erythriol-4P; IPP, isopentenyl diphosphate; DMAPP, dimethylallyl diphosphate; GGPP, geranylgeranyl diphosphate; CPP, copalyldiphosphate; entCPP, ent-copalyldiphosphate; GAs, Gibberellins; GGPPS, geranyl-geranyldiphosphate synthase; IDI, isopentenyl diphosphate isomerase; CPPS, copalyldiphosphate synthase; entCPPS, ent-copalyldiphosphate synthase; MiS, miltiradiene synthase; FS, ferruginol synthase; HFS, hydroxy-ferruginol synthase; C_20_-Ox, C_20_-oxidase.

The isoprenoid pathway has been largely targeted in plants and microorganisms to direct the metabolic flux of precursors and intermediates towards the biosynthesis of high-value terpenoids. In these studies, several enzymes and/or the availability of intermediates belonging to the MEP pathway have been reported to be limiting to the biosynthesis of various terpenoids (Nielsen and Keasling, 2016; Vickers et al., 2017). The ADs, like all other terpenes, are derived from the condensation of universal five-carbon (C5) units, isopentenyl diphosphate (IPP) and its allylic isomer dimethylallyl diphosphate (DMAPP). In higher plants, IPP and DMAPP are synthesized either by the cytosolic mevalonic acid (MVA) pathway (Newman and Chappell, 1999) or by the plastidial 2-methyl-d-erythritol-4-phosphate (MEP) pathway (Rohmer, 1999). Although a crosstalk between these pathways may occur (Bick and Lange, 2003; Schuhr et al., 2003; Hemmerlin et al., 2012), the downstream terpenoid biosynthetic steps are highly conserved in all terpenoid-synthesizing organisms, regardless of the origin of IPP and DMAPP building blocks. The synthesis begins with the repetitive addition of IPP units to a DMAPP precursor by sequential head-to-tail condensation reactions by the prenyl-transferases group of enzymes. This process generates linear prenyl pyrophosphate molecules that vary in chain length by 5-carbon units, and include the C10 geranyl pyrophosphate (GPP), C15 farnesyl pyrophosphate (FPP), and C20 geranylgeranyl pyrophosphate (GGPP). These pyrophosphates are then the immediate precursors to the various terpenes, which are synthesized by the action of terpene synthases. As such GPP is the precursor to monoterpenes, FPP to sesquiterpenes and triterpenes, and GGPP to diterpenes and tetraterpenes (**Figure 1**).

As mentioned earlier, the aethiopinone content is very low in roots of naturally grown *S. sclarea* plants. In the last few years we have developed a HR-based system and designed different complementary metabolic approaches to modify the metabolic flux towards a higher biosynthesis of this interesting AD in *S. sclarea* HRs.

Based on previous general knowledge of diterpene biosynthesis in angiosperms indicating that the first two enzymes of the MEP pathway, 1-deoxy-D-xylulose-5-phospate synthase (DXS) and 1-deoxy-D-xylulose-5-phosphate reducto-isomerase (DXR) are limiting for the biosynthesis of terpenoids, we overexpressed these two genes in *S. sclarea* HRs and found that the ectopic expression of *DXS* and *DXR* genes increased the AD content by 3–4 fold in *S. sclarea* HRs (Vaccaro et al., 2014; Vaccaro et al., 2019). The major role of these two enzymes in controlling the flux through the MEP pathway towards high-value terpenoids has been previously reported in different plant species (Wright et al., 2014; Banerjee and Sharkey, 2014; Rodriguez-Concepcion and Boronat, 2015).

Another crucial branch‐point enzyme for the biosynthesis of diterpenoids in plants is controlled by the geranylgeranyl pyrophosphate synthase (GGPPS), which catalyzes repetitive addition of IPP units in a sequence of elongation reactions to produce the 20 carbon diterpene precursor GGPP (Beck et al., 2013). GGPP is then shared by several biosynthesis pathways of plastid‐localized terpenoids, including the carotenoids and their derivatives, as well as the gibberellins, plastoquinones, tocopherols, chlorophylls, and diterpenes (Vranovà et al., 2013). Interestingly, we have previously reported that the *GGPPS* gene was up-regulated and the expression level was highly correlated with an enhanced content of aethiopinone, in *S. sclarea* HRs either upon elicitation (Vaccaro et al., 2017) or by overexpressing different WRKYs or MYC2 transcription factors (Alfieri et al., 2018), suggesting that this enzyme might be also limiting to boost the accumulation of aethiopinone in *S. sclarea* HRs, as found for several other plant terpenoids (Vranovà et al., 2013).

From GGPP, plant diterpenoids are produced through modular pathways with different combinations of diterpene synthases (diTPSs), cytochrome P450–dependent monooxygenases (P450s), and various other enzymes that increase structural diversity (Zi et al., 2014; Zerbe and Bohlmann, 2015; Tarkowskà and Strnad, 2018).

The biosynthetic routes of labdane-related diterpenes have been first and thoroughly described in resin acid biosynthesis in conifers/gymnosperms. The formation of diterpenes in conifer diterpene resin acids biosynthesis proceeds *via* the initial bicyclization of GGPP into (+)-copalyl diphosphate [(+)-CPP] at the class II active site of abietadiene synthase, a bifunctional diTPS. The (+)-CPP intermediate then translocates to the class I active site and undergoes secondary cyclization and further rearrangements *via* intermediate carbocations (Peters and Croteau, 2002; Peters, 2010; Hall et al., 2013). More recent studies have demonstrated that in species belonging to Lamiaceae the synthesis of labdane-related diterpenes also begins with the cyclization of GGPP to CPP, catalyzed by the copalyl diphosphate synthase (CPPS), a class II diTPS. The second committed step in the biosynthesis of this group of diterpenes is carried out by a kaurene synthase-like enzyme, called miltiradiene synthase (MiS), which convert CPP to miltiradiene. The oxidation of miltiradiene to abietatriene occurs spontaneously (Zi and Peters, 2013). One key intermediate downstream abietatriene is ferruginol. Several cytochrome P450 monooxygenases (CYP) of the CYP76 family from *S. miltiorrhiza*, *R. officinalis*, and *S. fruticosa*, able to convert abietatriene to ferruginol, have been characterized (Guo et al., 2013; Zi and Peters, 2013; Brückner et al., 2014; Cheng et al., 2014; Božić et al., 2015; Scheler et al., 2016; Ignea et al., 2016; Bathe et al., 2019). These enzymes are collectively called ferruginol synthases (FSs). *In vitro* characterization of one of these enzymes (CYP76AH4 from *R. officinalis*) indicated that abietatriene rather than miltiradiene is the substrate (Zi and Peters, 2013). It has also been shown that the previously identified FS from *S. fruticosa* and *R. officinalis* carries out not a single but two successive oxidations leading to the next intermediate 11-hydroxyferruginol and were renamed 11-hydroxyferruginol synthases (HFSs) (Scheler et al., 2016). The subsequent oxidation, at the C_20_ position of 11-OH-ferruginol, catalyzed by a C20-oxidase, leads to carnosic acid (**Figure 1**).

In *Salvia sclarea*, the most well characterized labdane type diterpene is sclareol, found in flowers. This compound, however, is not derived from CPP, but a Labd-13-en-8-ol diphosphate synthase (*Ss*LPPS) cyclizes GGPP in labda-13-en-8-ol diphosphate (LPP), which is subsequently converted into scareol (Caniard et al., 2012). As far as the ADs synthesized in *S. sclarea* and other *Salvia* spp roots, although they were identified and their chemical structures established more than two decades ago (Boya and Valverde, 1981; Ulubelen et al., 1997; Kuzma et al., 2006), their biosynthetic pathway is poorly understood. No data are available on a putative role of a CPPS in the first cyclization step of GGPP to CPP, which could serve potentially as substrate of subsequent enzymatic reactions to yield aethiopinone, 1-oxo-aethiopinone and salvipisone. However, an indirect evidence was provided by our elicitation and TF overexpression studies, which indicated that the expression level of the *CPPS* gene was highly correlated with the content of ADs in *S. sclarea* HRs (Vaccaro et al., 2017; Alfieri et al., 2018). Subsequent enzymatic steps from CPP to aethiopinone and salvipisone have not been elucidated, although, on a structural basis, they might be derived from ferruginol, as reported for other ADs in different Lamiaceae species. We hypothesize that abietaquinone, the oxidized form of 11-hydroxy-ferruginol, may be an intermediate in the biosynthetic route to aethiopinone, which may be oxidized to 1-oxo-aethiopinone or converted to salvipisone (**Figure 1**).

On the basis of our previous findings, in the present study we have targeted the *GGPPS* and *CPPS* genes to better understanding their contribution in the biosynthesis of aethiopinone and other ADs in *S. sclarea*, with the aim of generating a modular metabolic engineering toolbox for increasing their content in *S. sclarea* HRs. By blocking the lateral GGPP competing enzyme *ent*-copalyl-diphosphate synthase (entCPPS), involved in gibberellin biosynthesis, using either chemical enzymatic inhibition or RNA*i*-mediated gene silencing, we corroborate the notion that the GGPP pool is limiting for aethiopinone and other AD biosynthesis in *S. sclarea* HRs. In addition, by overexpressing the *GGPPS* and *CPPS* genes, individually or in combination, we also support the notion that these two enzymes constitute metabolic bottlenecks to obtain a significant increase in the biosynthesis of aethiopinone and other ADs in *S. sclarea* HRs.

## Materials and Methods

### Hairy Root Transformation and Growth Conditions

Axenic *S. sclarea* plants were obtained as described in Vaccaro et al. (2014) and grown at 23°C under a photoperiod of 8 h dark and 16 h light (110 μmol m^-2^ s^-1^) in a controlled growth chamber. Control (empty vector, EV) or transformed *S. sclarea* HRs were obtained by infection of leaf discs from *S. sclarea* axenic plantlets with *Agrobacterium rhizogenes* ATCC 15835 strain (purchased at the American Type Culture Collection, www.atcc.org), as previously described in Vaccaro et al. (2014; 2017). Stable control and transformed HR lines were maintained in liquid hormone-free MS medium in the dark under continuous agitation (150 rpm). HR growth was assessed by inoculating equal amounts (5 g fresh weight) of control or transgenic HR lines into 1 L of MS hormone-free liquid medium and dry weight (DW) was monitored for 1 month at 1-week intervals.

### Chemical Inhibition and RNAi Silencing of Ent-Copalyl Diphosphate Synthase

Equal amounts of *S. sclarea* HRs, sub-cultured in MS hormone-free medium for 3 weeks, were treated for 30 days with 100 μM 2-chloroethyl-N,N,N-trimethylammonium chloride (CCC), a known inhibitor of the ent-copalyl diphosphate synthase (entCPPS), or with an equal volume of H_2_O as mock control.

The competitive gibberellin biosynthetic route was also inhibited by RNA interference (RNAi) of the *entCPPS* gene. The full-length coding sequence of the *S. sclarea entCPPS* gene was obtained by using a set of degenerate primers (**Table S1**), designed on conserved regions of entCPPS proteins of different plant species. A partial sequence (expected size of 518 bp) of this gene was amplified from *S. sclarea* root cDNA (**Figure S1A**) and cloned into pCR2.1 vector (Invitrogen, Carlsbad, CA, USA). Three random positive clones were sequenced and identical to each other, showing an identity at the amino acid level higher than of 70% to the coding sequence of the *A. thaliana entCPPS* orthologue gene (**Figure S1B**). Using this partial sequence, a specific primer was designed for extending the coding sequence by 3′-RACE PCR (**Figure S1C**). An amplicon of 1821 bp was obtained, sequenced and the sequence deposited in GenBank (Accession MK517475). This sequence corresponds to the portion of the deduced protein from the amino acid in position 200 to the stop codon for *entCPPS*. The alignment of this amplicon with the entCPPS amino acid sequence of other plant species identified the presence of functional domains, such as the aspartate-rich “DIDD” box, which is responsible for the synthase activity (**Figure S1D**). The cDNA pool of the *S. sclarea* roots was used as a template to amplify a short fragment (450 bp) of the 3′ coding sequence, which was sequenced, and then inserted by recombination between the *att*B1/*att*B2 and *att*B2/*att*B1 recombination sites into the pHELLSGATE12 binary vector, kindly provided by CSIRO (*Commonwealth Scientific and Industrial Research Organisation)* (**Figure S1E**). The vector was transferred into the *A. rhizogenes* ATCC15834 strain and the recombinant plasmid used for transformation of *S. sclarea* HRs, according to Vaccaro et al. (2014). Several kanamycin resistant hairy root lines putatively silenced were characterized for the presence of the *neomycin phosphotransferase II* (*nptIII*) and the *protein-tyrosine phosphatase* (*rolB*) gene. Absence of contaminating bacteria was confirmed by negative amplification of the bacterial *virD2* gene. *A. rhizogenes* transformed with the empty RNA*i* vector was used as PCR positive control (C^+^) (**Figure S1F**). Three independent putatively silenced HR lines were analyzed for the transcript level of the endogenous *entCPPS* gene and used for metabolic analyses of the AD content.

### Identification and Cloning of *S. sclarea GGPPS* and *CPPS* cDNAs

*S. sclarea* roots were homogenized in liquid nitrogen and total RNA extracted with the plant RNA purification kit (Norgen Biotek Corporation Ontario, Canada) according to the manufacturer’s instructions. The complementary DNA (cDNA) was synthesized from 1 µg of total RNA treated with RNase-free DNAse I (Invitrogen, Carlsbad, CA, USA), using Superscript III Reverse transcriptase (Invitrogen). The partial sequences of *S. sclarea GGPPS* and *CPPS* genes were obtained by PCR amplification with High Fidelity DNA Polymerase (Pfx, Invitrogen, Carlsbad, CA, USA) using different combinations of primers designed on the basis of conserved regions identified from the alignment of GGPPS and CPPS proteins of different plant species available in GenBank (**Table S1**). The obtained amplified fragments were cloned into pCR2.1 vector using TA-Cloning kit (Invitrogen), sequenced, and identified as the partial fragments of *S. sclarea GGPPS* and *CPPS* cDNA corresponding to 450 bp and 700 bp, respectively. Using these partial sequences, specific primers were designed for extending the coding sequence with 3′- and 5′-RACE PCR using SMART™ RACE cDNA Amplification Kit (Clontech Laboratories Inc, CA, USA). The fragments obtained were cloned into pCR2.1 vector using the TA-Cloning kit (Invitrogen) and sequenced. The full-length coding sequence of *S. sclarea GGPPS* (GeneBank Accession MK442922) corresponds to 1182 bp and the full-length *S. sclarea CPPS* (GeneBank Accession MK442923) corresponds to 2574 bp. The deduced amino acid sequences were aligned to other available plant GGPPS and CPPS sequences, to identify the presence of the plastidial signal peptide and functional domains, such as the aspartate-rich DIDD or DD(X)D box (**Figures S2** and **S3**).

### Plasmid Construction for Overexpressing *GGPPS* and *CPPS* Genes in *S. sclarea* HRs

Specific primers (**Table S1**) containing the short *CACC* sequence at the 5′ were used for amplifying the *S. sclarea GGPPS* and *CPPS* cDNAs by using a High Fidelity DNA Polymerase (Pfx, Invitrogen, Carlsbad, CA, USA), which were inserted into pENTR/kit D-TOPO^®^ (Invitrogen) to generate an *Entry-Clone*. After verifying the correct insertion and the absence of mutations by sequencing, the coding sequences of the two genes were subcloned downstream the constitutive viral 35SCaMV promoter through the LR reaction (*The Gateway*^®^ *LR Clonase*™ *enzyme mix kit*, *Invitrogen*) into the Gateway binary vector pK7WG2D (http://www.psb.ugent.be/gateway/).

For harboring vectors co-expressing the two genes, the full-length *GGPPS* cDNA was amplified by High Fidelity DNA Polymerase (Pfx, Invitrogen, Carlsbad, CA, USA) by using specific primers containing the recombination sites attB3 and attB4 for cloning into the Gateway pDONR P4-P3 vector. The coding sequence of *CPPS* was amplified using specific primers with the recombination sites attB1 and attB2 and cloned into the Gateway pDONR221 vector (**Table S1**). The amplicons for both genes were cloned into the Gateway pDONR vectors using the BP recombinase (Invitrogen, Carlsbad, CA, USA). Correct insertion and the absence of mutations were verified by sequencing and *GGPPS* and CPPS cDNAs were cloned independently into the Gateway binary vector pK7M34GW2-8M21GW3D (http://www.psb.ugent.be/gateway/), under the control of the constitutive promoters p*35S* (*GGPPS*) or pr*olD* (*CPPS*). Specific primers were used to select streptomycin and spectinomycin resistant (*Sm/Spr^+^*) colonies containing plasmids with both genes (**Table S1**). The vectors were then shuttled into *A. rhizogenes* strain ATCC15834, and used for transformation of *S. sclarea* axenic leaf sections to obtain transgenic *S. sclarea* HRs, according to Vaccaro et al. (2014). Control HR lines were obtained by transforming *A. rhizogenes* carrying the pK7WG2D vector harboring a 174 bp fragment of *A*. *thaliana GUS* gene.

### RNA Extraction, RT-PCR, and qRT-PCR Analysis

Ten kanamycin-resistant HR lines putatively overexpressing the *GGPPS* and *CPPS* genes individually or in combination were firstly screened for the genomic insertion of the *neomycin phosphotransferase II* (*NPTII*) and the *protein-tyrosine phosphatase* (*rolB*) gene by PCR and the absence of bacterial DNA by targeting the bacterial virD2 gene (**Figure S4**), using specific primers (**Table S1**). Total RNA was extracted using the plant RNA/DNA Purification kit (Norgen Biotek Corporation Ontario, Canada), according to the manufacturer’s protocol. For semi-quantitative RT-PCR, complementary DNA was synthesized from 1 μg total RNA treated with RNase-free DNase I (Invitrogen, Carlsbad, CA, USA), using random hexamers and the Superscript III reverse transcriptase (Invitrogen, Carlsbad, CA, USA) at 50°C for 50 min. In the PCR reactions, 1 μl of cDNA was used as template with specific primers (**Table S1**) and 2.5 units of Taq polymerase (Invitrogen, Carlsbad, CA, USA). The *S. sclarea 18S* gene-specific primers were used as reference internal gene.

Quantitative RT-PCR was performed using a Light Cycler system (Roche Diagnostics Ltd, Lewes, UK) according to the manufacturer’s instructions. Reactions were performed in a 20 μl volume with 0.5 μM primers and Light Cycler-DNA Master SYBR Green I mix (Roche Diagnostics Ltd, Lewes, UK). Gene-specific primers (**Table S1**) for qRT-PCR were used to analyze the relative expression level of the genes compared to the internal reference gene *SsActin* (JZ923579), using the relative quantitative analysis method (2^-ΔΔCT^). Gene expression analyses were performed in triplicate on three independent HR lines overexpressing *GGPPS* and *CPPS* individually or in combination and compared to control HR lines.

### HPLC-DAD Analysis of Abietane Diterpenes

Lyophilized and powdered plant tissues from control, CCC-treated or transformed HR lines were extracted with acetone for 72 h at room temperature, as previously described (Vaccaro et al., 2014). Briefly, the extracts were evaporated under reduced pressure, the residues dissolved in methanol and loaded (50 μl) on a C8 column (Agilent, ZORBAX Eclipse C8 250 × 4.6 mm) for targeted high-performance liquid chromatography-diode-array detector (HPLC-DAD) analysis (Agilent 1200 Series, G1312A binary pump, G1329A automatic sample injector, G1315D diode array detector). The targeted ADs were detected at 280 nm and their concentration calculated by interpolation of the peak areas on calibration curves obtained using standard purified compounds over the range of 10–200 μg ml^-1^. Content of ADs in roots was expressed as mg g^-1^ of HR dry weight (DW) in small scale cultures (100 ml) or as mg L^-1^ in 1 L of HR culture. Chemical structures of the most abundant *S*. *sclarea* ADs and typical chromatographic patterns for control and representative transformed HR lines are reported in **Figure S5**.

### Statistical Analysis

The data for the HR growth, metabolic, and transcriptional analyses are represented as the mean ± sd of at least three independent biological experiments performed in triplicate either for transformed HR lines or control HR line. Significant differences in the gene transcript levels were statistically analyzed by one-way analysis of variance (ANOVA), with Tukey’s *post-hoc* test, while differences in the HR dry weight and AD content were examined by the two-way analysis of variance (ANOVA) with Bonferroni *post-hoc* test analysis, using GraphPad Prism 5 software. Statistically significant threshold was fixed at *P* ≤ 0.05, *P* ≤ 0.01, or *P* ≤ 0.001.

## Results

### Inhibition of the GGPP Competitive Gibberellin Lateral Route and ADs Content in *S. sclarea* HRs

**Figure 2:**
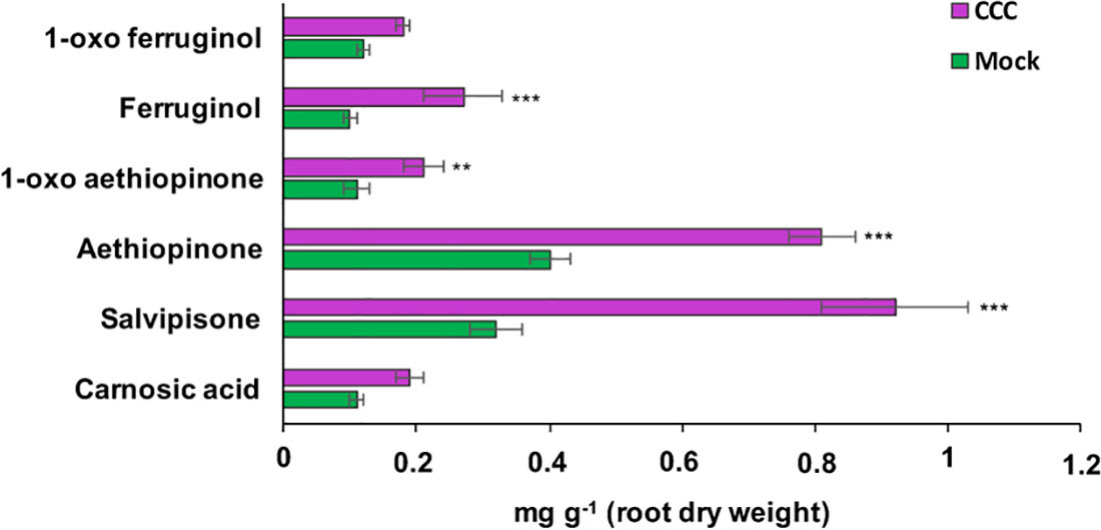
Content of the targeted abietane diterpenes determined by HPLC-DAD, in *S. sclarea* hairy root lines treated for 30 days with 2-chloroethyl-N,N,N-trimethyl-ammonium chloride (CCC), a known inhibitor of the lateral gibberellin biosynthesis route form GGPP. Values of abietane diterpene content are means of three biological replicates ± SD for each HR line. ** and *** denote statistical significance at P ≤ 0.01 and P ≤ 0.001, respectively, between the treated and untreated control HR lines.

**Figure 3:**
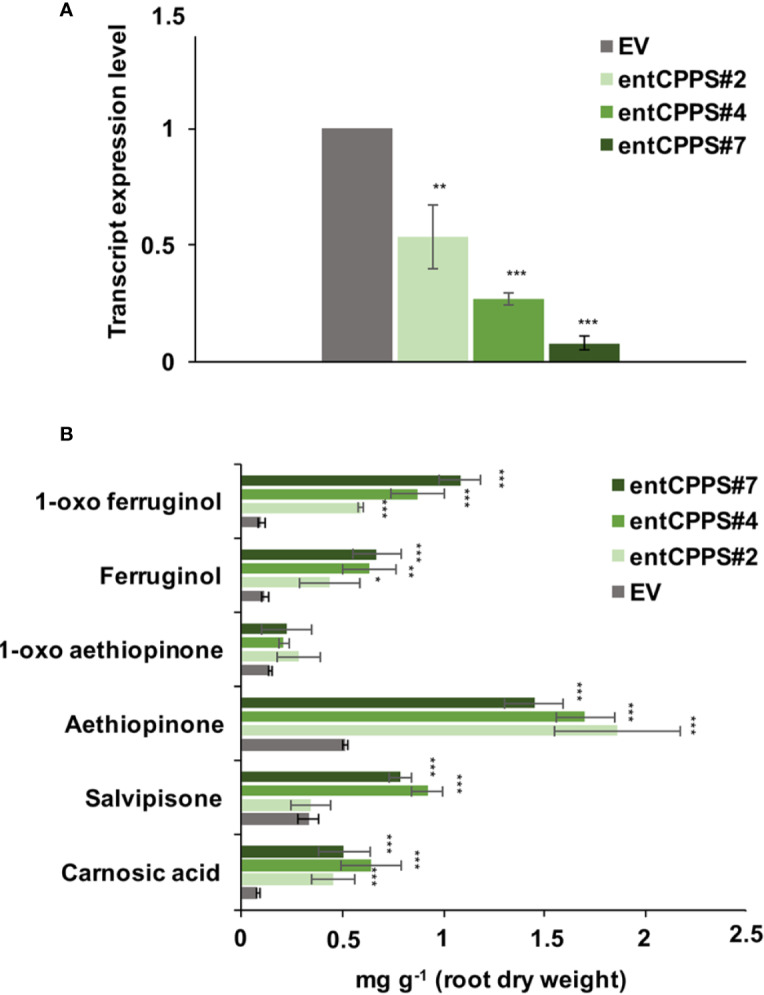
Silencing of *entCPPS* in hairy root lines. **(A)** Transcript level of *entCPPS* (accession n. MK517475) in independent silenced HR lines determined by qRT-PCR are expressed as relative fold-change compared to the endogenous level in control HR lines transformed with the empty vector (EV). The values were normalized using actin as internal control. **(B)** Content of the analyzed abietane diterpenes determined by HPLC-DAD. Values of the transcript level and abietane diterpene content are means of three biological replicates ± SD for each HR line. ** and *** denote statistically significant differences between the transgenic and control HR lines, at P ≤ 0.01 and P ≤ 0.001, respectively.

ADs are synthesized in the plastid from GGPP, which is also the precursor for gibberellins. The branch point from GGPP to gibberellins is enzymatically controlled by the *ent*-copalyl-diphosphate synthase (entCPPS), a class II diterpene synthase. To increase GGPP availability for aethiopinone biosynthesis, this lateral competing route was inhibited by treating *S. sclarea* HRs for thirty days with 100 μM CCC, a known chemical inhibitor of entCPPS enzyme activity (Hedden et al., 2010). The total AD content in CCC-treated hairy roots (2.52 mg g^-1^ DW) was 2.2-fold higher than control HRs (1.15 mg g^-1^ DW). The content of aethiopinone, 1-oxo-aethiopinone, and salvipisone, as well as of other ADs (carnosic acid, ferruginol, 1-oxo-ferruginol) in CCC-treated HRs was significantly enhanced (P ≤ 0.001) compared to the control untreated HRs, with the most relevant effect on the content of aethiopinone (2-fold increase) (**Figure 2**). We also silenced the *entCPPS* gene by RNA, and identified HR lines with reduced expression levels of the endogenous gene ranging from 60% (silenced line #2) to 90% (silenced line #7) (**Figure 3A**). Depending on the level of silencing of the endogenous *entCPPS*, in the silenced HR lines, the total AD content increased significantly, in the range of 3.97–4.98 mg g^-1^ HR DW. Compared to the control HR line (0.52 mg g^-1^ DW), a consistent 3.5-fold increase was observed for aethiopinone (1.86 mg g^1^ DW) in the silenced HR line #2, which had the highest reduction in the expression level of the endogenous gene. Interestingly, also the content of carnosic acid, salvipisone, and 1-oxo-ferruginol enhanced by 7-, 2.5-, and 9-times, respectively, compared to the content in the control HR line (**Figure 3B**). Interestingly, inhibition of the GAs lateral route either chemically or by RNAi did not cause any growth inhibition of *S. sclarea* HRs (**Figures S6A, B**). The expected negative effects due to low GAs levels might be minimized in HRs, since they are adventitious roots which differentiate through a modified balance of auxins/cytokinins, due to the insertion of *rol* genes in the plant genome. These set of data indicate that the accumulation of aethiopinone and other ADs in *S. sclarea* HRs benefits from an indirect higher GGPP availability, generated by blocking the GAs competitive lateral route.

### Overexpressing *GGPPS* Increases the Content of Abietane Diterpenes in *S. sclarea HRs*

**Figure 4:**
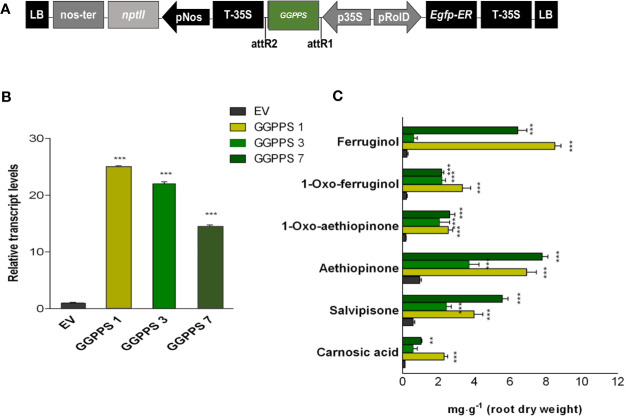
*S. sclarea* hairy root lines overexpressing the *GGPPS* gene. **(A)** Map of T-DNA of *GGPPS* construct used for HR transformation. **(B)** Transcript level of *GGPPS* gene (accession n. MK442922) in transgenic HR lines determined by qRT-PCR are expressed as relative fold-increase compared to the endogenous level in control HR lines transformed with the empty vector (EV). The values were normalized using *18S* gene as control (accession n. DQ667222). **(C)** Content of the analyzed abietane diterpenes determined by HPLC-DAD. Values for transcript level and abietane diterpene content are means of three biological replicates ± sd for each HR line. ** and *** denote statistically significant differences between the transgenic and control HR lines, at P ≤ 0.01 and P ≤ 0.001, respectively.

The above reported evidence for potential limited GGPP pool in the metabolic route to abietane diterpenes, together with our previous results on a significant correlation between the expression level of *GGPPS* gene and AD accumulation either by elicitation or by TF overexpression in *S. sclarea* HRs (Vaccaro et al., 2017; Alfieri et al., 2018), suggests that GGPPS might be a potential target for boosting the biosynthesis of ADs in *S. sclarea* HRs. Therefore, assuming that the IPP and DMAPP, the immediate GGPP precursor, would not be limiting, the full-length *GGPPS* gene, including the plastid transit peptide (**Figure 4A**), was constitutively overexpressed in *S. sclarea* HRs. Several independent transformed HR lines were obtained and analyzed by PCR amplification of genomic DNA using specific primers for *NPTII* and *rolB* genes (**Figure S4**). Three independent transformed HR lines with varying transcript levels of the *GGPPS* gene, assessed by q-RT-PCR (**Figure 4B**) were further analyzed for metabolic changes in the ADs. The overexpression of the *GGPPS* gene significantly increased the content of all analyzed ADs in *S. sclarea* HRs (**Figure 4C**). The content of aethiopinone (7.80 ± 0.71 mg g^-1^ DW), salvipisone (5.57 ± 0.78 mg g^-1^ DW), and ferruginol (8.51 ± 0.35 mg g^-1^ DW) were approximately 8-, 7-, and 28-times, respectively, higher than that in the control HR line (0.99 ± 0.08 mg g^-1^ DW). These data support further our hypothesis that the GGPP pool in *S. sclarea* HRs is limiting for the biosynthesis of ADs and indicate that *GGPPS* overexpression is an efficient strategy to increase the overall content of ADs in *S. sclarea* HRs.

### CPPS Contributes Also to the Accumulation of Abietane Diterpenoids in *S. sclarea HRs*

**Figure 5:**
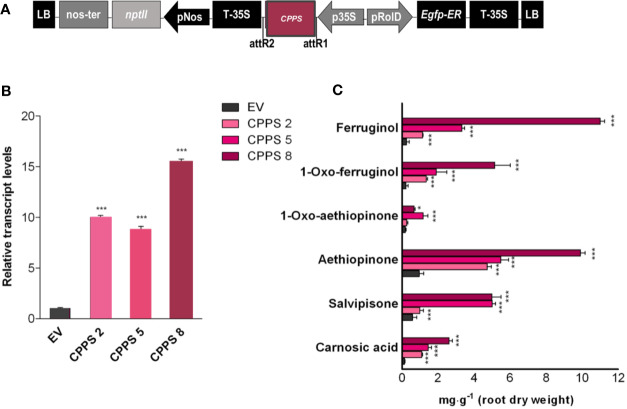
*S. sclarea* HR lines overexpressing the *CPPS* gene. **(A)** Map of T-DNA construct used for HR transformation. **(B)** Transcript level of *CPPS* gene (accession n. MK442923) in transgenic HR lines determined by qRT-PCR are expressed as relative fold-increase compared to the endogenous level in control HR lines transformed with the empty vector (EV). The values were normalized using *18S* gene as control. **(C)** Content of the targeted abietane diterpenes determined by HPLC-DAD. Values for transcript level and abietane diterpene content are means of three biological replicates ± sd for each HR line. ** and *** denote significant statistical differences between the transgenic and control HR lines, at P ≤ 0.01 and P ≤ 0.001, respectively.

Based on data already reported for other cyclic diterpenoids, we hypothesized that the initial biosynthetic step of aethiopinone and other abietane-type diterpenoids found in *S. sclarea* roots might start with the conversion of the GGPP to copalyl diphosphate (CPP), an enzymatic reaction catalyzed by the copalyl diphosphate synthase (CPPS) belonging to a class II diterpene synthase (class II diTPSs) (**Figure 1**). Our previous results have also indicated a tight correlation between the level of *CPPS* gene expression and the content of ADs upon elicitation with methyl jasmonate (Vaccaro et al., 2017) or by overexpressing different *WRKY* transcription factors (Alfieri et al., 2018). To prove whether or not CPPS is also limiting in the biosynthetic route of ADs in *S. sclarea*, the full-length *SsCPPS* gene, including the transit peptide (**Figure 5A**) were constitutively overexpressed in *S. sclarea* HRs (**Figure 5A**). Several independent transformed HR lines analyzed by PCR amplification on genomic DNA using specific primers for the *NPTII* and *rolB* genes (**Figure S4**). Three independent HR lines, overexpressing the different levels of *CPPS* gene (**Figure 5B**) were analyzed further for their AD metabolic profiles. Similarly to what we found in the *GGPPS* overexpressing HR lines, the *CPPS* overexpression generally boosted the metabolic flux towards a higher AD content (**Figure 5C**), with a significant 10-fold increase in the aethiopinone content (10.44 ± 0.21 mg g^-1^ DW, P ≤ 0.001) compared to the control HR lines (0.99 ± 0.08 mg g^-1^ DW). Additionally, the content of ferruginol (30-fold, 11.50 ± 0.38 mg g^-1^ DW) and salvipisone (9-fold, 6.65 ± 0.72 mg g^-1^ DW) was also enhanced significantly by overexpressing the *CPPS* gene in *S. sclarea* HRs.

Taken together, these data indirectly suggest that the biosynthesis of aethiopinone in *S. sclarea* might start from the conversion of GGPP to CPP by CPPS, as reported for other ADs in several other plant species. Furthermore, the biosynthesis of aethiopinone and other ADs in *S. sclarea* HRs seems to be limited by the levels of CPP, since the overexpression of *CPPS* can overcome this metabolic bottleneck and enhance the accumulation of ADs.

### Co-Expression of *GGPPS* and *CPPS* and Abietane Diterpene Content in *S. sclarea* Hairy Roots

**Figure 6:**
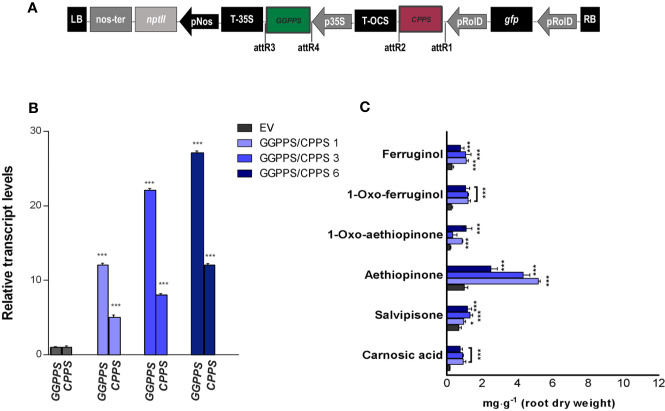
*S. sclarea* HRs co-overexpressing the *GGPPS* and *CPPS* genes. **(A)** Map of T-DNA construct used for HR transformation, where the *GGPPS* coding sequence was cloned downstream of the 35S promoter and *CPPS* coding sequence under the rolD promoter. **(B)** Transcript level of *GGPPS* and *CPPS* genes in transgenic HR lines determined by qRT-PCR are expressed as relative fold-increase compared to the endogenous level in control HR lines transformed with the empty vector (EV). The values were normalized using *18S* gene as control. **(C)** Content of the analyzed abietane diterpenes determined by HPLC-DAD. Values of transcript level and abietane diterpene content are means of three biological replicates ± sd for each HR line. ** and *** denote statistically significant differences between the transgenic and control HR lines, at P ≤ 0.01 and P ≤ 0.001, respectively.

Following our positive results in enhancing AD content by individually overexpressing *GGPPS* and *CPPS* genes, we co-expressed the two genes in trying to enhance further the content of ADs in *S. sclarea* HRs (**Figure 6A**). The *GGPPS* and *CPPS* transcript levels upon co-expression in three independent HR lines are reported in **Figure 6B**. An increase in the accumulation of aethiopinone and other ADs was triggered in all the HR lines co-expressing *GGPPS* and *CPPS* (**Figure 6C**), with the highest boosting effect on aethiopinone accumulation. It is worth noting that, however, the effect of the gene co-expression on the increase in aethiopinone did not appear to be additive, since the maximum increase in aethiopinone was detected in the HR line *GGPPS/CPPS #1* (6-times higher than the content in the control HR line), characterized by the lowest transcript level of both genes. The weaker boosting effect on aethiopinone accumulation in the other two co-expressing HR lines might be due to unpredictable metabolic blocks, such as an impaired translation of simultaneous high level of *GGPPS* and *CPPS* transcripts, causing a potential unbalanced ratio between the levels of the two enzymes, and/or relative substrates, or product mediated feedback inhibition of enzyme activity. Further experiments on GGPPS and CPPS protein levels, enzymatic activity measurements, or determination of the GGPP and CPP content are necessary to ascertain this possibility, in order to select the best performing co-expressing HR lines in terms of accumulation of aethiopinone.

### HR Growth and Final Yield in Abietane Diterpenes in Engineered *S. sclarea* HRs

Perturbation of the metabolic flux by gene engineering in plants can often result in growth defects, owing to the accumulation of toxic intermediates and/or negative feedback, which would produce undesirable pleiotropic effects on the final yield of the desired product (Leone et al., 2007; Li et al., 2016). The best performing transgenic HR lines, in terms of aethiopinone content, overexpressing the two genes individually or in combination, were grown in a scaled-up culture (1 L) and HR dry weight monitored weekly. As expected, some growth detrimental effects were observed in the transgenic HR lines; however, lines *GGPPS #7*, *CPPS#8*, and *GGPPS/CPPS #3* showed negligible growth impairment compared to the control line (**Figures S6 C–E**). Analysis of the individual compounds and of the final AD yield at the end of a 4-week growth period (**Table 1**), revealed that in *S. sclarea* HRs the overexpression of *CPPS* gene was the most efficient strategy in enhancing the content of aethiopinone and other ADs. In particular, the final yield of aethiopinone was 208.98 ± 2.18 mg L^-1^ and that of ferruginol was 230.40 ± 3.74 mg L^-1^, significantly higher than the content in the control line (19.82 ± 2.23 and 6.01 ± 0.92, respectively).

**Table 1:** Yield of individual abietane diterpenoids (ADs) and final total yield, expressed as mg L^-1^ in engineered *S. sclarea* hairy root lines, overexpressing the *GGPPS* and *CPPS* genes individually or in combination, after 30 days of growth in 1 L liquid medium.

HR lines	Carnosic acid	Salvipisone	Aethiopinone	1-oxo-aethiopinone	1-oxo-ferruginol	Ferruginol	Final total AD yield
			mg L^-1^				
**Control**	3.60 ± 0.50	13.83 ± 1.23	19.82 ± 2.23	3.86 ± 0.63	5.31 ± 1.15	6.01 ± 0.92	52.41 ± 3.20
***GGPPS* 7**	20.74 ± 1.50**	111.34 ± 7.50***	155.84 ± 7.20***	52.76 ± 3.70***	43.66 ± 4.50***	128.64 ± 8.20***	512.91 ± 12.31***
***CPPS 8***	62.36 ± 3.89**	133.18 ± 7.80***	208.98 ± 2.18***	13.38 ± 0.82***	20.34 ± 4.32*	230.40 ± 3.74***	750.98 ± 16.50***
***GGPPS/CPPS 1***	18.46 ± 1.65**	19.23 ± 1.26***	103.66 ± 1.95***	17.24 ± 0.60***	24.18 ± 1.32	22.06 ± 1.13***	204.86 ± 6.18***

The data represent the mean values ± SD of three technical repeats from two biological replicates of transgenic lines showing negligible growth impairment (GGPPS 7, CPPS 8, and GGPPS/CPPS 1).

** and *** denote statistically significant differences between the transgenic and control HR lines at P ≤ 0.01 and P ≤ 0.001, respectively.

## Discussion

In the last few years our interest has been focused on ADs synthesized in *S. sclarea* roots, particularly aethiopinone, which has promising anti-proliferative activities in several solid tumor lines (Vaccaro et al., 2014) and, as previously reported, in leukemia cells (Rozalski et al., 2006). However, the possibility of using this compound in the discovery of novel antitumor drug is strongly restrained by the low amount extractable from natural plant sources. Diterpene biosynthesis, and, more in general, bioactive plant-derived secondary metabolites, is limited by several metabolic bottlenecks, such as insufficient precursor supply, flux of precursors into competing pathways, low enzyme levels and activity, and toxic or inhibitory effects of intermediates (Guerriero et al., 2018). However, different and complementary metabolic engineering and elicitation strategies have been successfully applied to prompt the accumulation of interesting bioactive plant-derived diterpenes, as demonstrated for the accumulation of tanshinones, a class of abietane-type diterpenoid quinone compounds in *Salvia miltiorrhiza*, (Kai et al., 2011a; Cheng et al., 2013; Shi et al., 2016; Wei et al., 2019).

We have previously demonstrated that it is feasible to enhance significantly by 4-times the accumulation of aethiopinone and other ADs in n *S. sclarea* HR axenic lines by overexpressing the *DXS* and *DXR* genes (Vaccaro et al., 2014; Vaccaro et al., 2019), encoding the first two committed enzymes of the plastidial terpenoid MEP-derived pathway (Estévez et al., 2001; Carretero-Paulet et al., 2006).

The biosynthetic route of aethiopinone from GGPP, the well-established common precursor of all plant diterpenes, has not been elucidated. However, to expand our knowledge on possible additional metabolic bottlenecks in the biosynthetic pathway of *S. sclarea* ADs, in this study we have designed two different approaches to boost the synthesis of ADs in *S. sclarea* HRs: i) increasing the availability of GGPP, the common precursor of diterpenes by inhibiting the GA competitive lateral route or overexpressing the *GGPPS; ii)* overexpressing the *CPPS* gene, for which we have found previously that the level of expression was highly correlated to an increase in the content of aethiopinone and other ADs.

### GGPP Availability Limits Accumulation of ADs in *S. sclarea* HRs

Modifying the allocation of GGPP among the different downstream metabolic branches has been postulated as a possibility to divert this common isoprenoid precursor towards the higher biosynthesis of a targeted end product in the plastids (Vranovà et al., 2013). The gibberellins are derived from plastidial GGPP pathway, which makes them a competitive branching route of diterpenoids. Our results have shown that significant accumulation of abietane diterpenes, primarily aethiopinone (a content 4-times higher than that of the control line), can be obtained by treating *S. sclarea* HRs with 2-chloroethyl-N,N,N-trimethyl-ammonium chloride (CCC), a known inhibitor of entCPPS enzymatic activity (Hedden et al., 2010). To increase the availability of GGPP, we also inhibited the *ent-CPPS* gene, acting at GGPP lateral gibberellin biosynthesis route, by RNA*i*-mediated silencing. This approach has been successful in increasing the content of different terpenoids in several plant species. A gain in artemisinin content was obtained in *Artemisia annua*, by down-regulating the competitive branch pathway genes β-caryophyllene synthase (*cps*), β-farnesene synthase (*bfs*), germacrene A synthase (*gas*), and squalene synthase (*sqs)* (Zhang et al., 2009; Lv et al., 2016). In *Nicotiana benthamiana*, the RNA*i*-mediated silencing of the endogenous 5-*epi*-aristolochene synthase (*eas*) and *sqs* genes, which encode enzymes that pulled on the FPP pool, resulted in a 2.8-fold increase in the accumulation of the sesquiterpene (+)-valencene (Cankar et al., 2015). Similarly, data presented here demonstrate that RNA*i*-mediated silencing of the *entCPPS* gene also contributes to an increase in ADs in *S. sclarea* HRs. This finding was further proved by the significant increase in total AD content, especially in aethiopinone accumulation (an 8-fold increase compared to the content found in the control line) by ectopically over-expressing the *GGPPS* gene. Altogether, these data suggest that the availability of GGPP might be also limiting for the biosynthesis of aethiopinone and other ADs in *S. sclarea* HRs, and are consistent with previous studies reporting that the overexpression of *GGPPS* enhances the content of other diterpenes, such as forskolin in *Coleus forskohlii (*Engprasert et al., 2004), taxol in *Arabidopsis thaliana* (Besumbes et al., 2004) and yeast (Engels et al., 2008), and tanshinone in *Salvia miltiorrhiza* (Kai et al., 2011a; Shi et al., 2016).

Although we have not measured the GGPP content in HR lines overexpressing the *GGPPS* gene, these complementary approaches reveal indirectly that increasing the GGPP pool, by inhibiting the GAs competitive lateral route or by additional GGPPS enzymatic units, is an efficient approach to boost the synthesis of ADs in *S. sclarea* HRs.

### CPPS Is Involved in ADs Synthesis and Accumulation in *S. sclarea* HRs

In Lamiaceae species, several cyclic diterpenes derive from copalyl diphosphate (CPP), which is formed by the protonation-initiated cyclization of GGPP, reaction catalyzed by the CPPS enzyme, as already demonstrated in different plant species accumulating different type of ADs (Gao et al., 2009; Caniard et al., 2012; Guo et al., 2013; Božić et al., 2015; Ignea et al., 2016; Scheler et al., 2016; Bathe et al., 2019). Thus far, there is no previous direct evidence of the involvement of this enzyme in the biosynthesis of aethiopinone and other ADs in *S. sclarea*. The alignment of deduced amino acid sequence of the full-length cDNA obtained from *S. sclarea* HRs evidenced a high identity with known class II diTPS, including also the *S. sclarea* Labd-13-en-8-ol diphosphate synthase (LPPS), the enzyme leading to the synthesis of sclareol from GGPP in the *S. sclarea* flowers, and CPPS of other species. In particular, SsCPPS showed the highest similarity with *Salvia miltiorrhiza* CPPS (89%), *Rosmarinus officinalis* (64%), and *Salvia fruticosa* (62%) and only 59% with LPPS of *S. sclarea* (**Figure S3**).

We have found that the expression level of the *CPPS* gene is also highly correlated (r^2^ = 0.99) with aethiopinone accumulation either in elicited *S. sclarea* HRs (Vaccaro et al., 2017) or in WRKY and MYC2 overexpressing HR lines (Alfieri et al., 2018). Overexpressing the *CPPS* gene resulted in a general increase in aethiopinone and other ADs in *S. sclarea* HRs, which was 10-fold higher than that in the control HR line, pointing to this enzyme as another enzymatic bottleneck for aethiopinone accumulation. Taken together these findings also provide a first indirect evidence on the involvement of a CPPS in the first step of GGPP protonation-initiated cyclization step leading to CPP as a possible intermediate precursor of aethiopinone in *S. sclarea*. A more direct evidence of the *CPPS* gene being involved in the aethiopinone biosynthesis might be provided by RNA*i*-mediated suppression of this gene, as shown with the tanshinone diterpenes in *S. miltiorrhiza* (Kai et al., 2011a) as well as by further studies on the purified enzyme, enzymatic activity and labeled precursor feeding experiments.

### Co-Expression of GGPPS and CPPS Genes and ADs Accumulation

Although the overexpression of a single gene encoding a key enzyme may contribute to higher availability of a limiting intermediate, frequently there are other limiting enzymatic steps contributing to the amount of the targeted metabolite. Therefore, regulation of two or multiple genes would be more suitable to achieve significant gains in product accumulation.

Co-expression of rate-limiting biosynthetic genes has been successfully applied to boost the synthesis of a variety of high-value plant-derived compounds. In *Catharanthus roseus* a high accumulation of monoterpenoid indole alkaloids was achieved by the simultaneous expression of *dxs* and geraniol-10-hydroxylase (*g10h*) or anthranilate synthase (*As*) genes (Peebles et al., 2010*)*. Co-activation of putrescine-*N*-methyltransferase (*pmt*) and tropinone reductase I (*trI*) effectively enhanced the yields of tropane alkaloids in *Anisodus acutangulus* HRs (Kai et al., 2011b), and in *Ophiorrhiza pumila* overexpression of *g10h* and strictosidine synthase (*str*) boosted camptothecin accumulation (Cui et al., 2015). As far as diterpenes, co-expression of *SmHMGR* and *SmGGPPS* (Kai et al., 2011a), *SmHMGR* and *SmDXR* (Shi et al., 2014), or *SmGGPPS a*nd *SmDXSII* (Shi et al., 2016) in *S. miltiorrhiza* HRs significantly improved the production of tanshinones.

Starting from the significant increase in aethiopinone we have obtained by overexpression of *GGPPS* and *CPPS* genes individually, we designed a “push and pull” strategy based on the simultaneous expression of these two biosynthetic genes in *S. sclarea* HRs. Compared to the basal content in the control HR line, the *GGPPS* and *CPPS* co-expression in *S. sclarea* HRs enhanced the aethiopinone content by 6-times, against the 8- and 10-fold increase triggered by over-expressing the two genes individually. However, in the co-expressing HR lines the increase in aethiopinone content was not strictly correlated to the level of expression of the individual genes. This might be probably due to an unexpected metabolic block, such as an impaired translation of the simultaneous high level of *GGPPS* and *CPPS* transcripts causing a potential unbalanced ratio between the levels of the two enzymes and/or relative substrates. These aspects underline the need of more accurate additional studies on the level of GGPP and CPP as well as on the relative enzymatic activities to select *S. sclarea* co-expressing HR lines with an unbiased ratio of the two enzymes and relative substrates, which could result in an additive or synergistic effect on the final yield of aethiopinone.

Our primary aim was to obtain high amount of aethiopinone for further characterization of its pharmacological and molecular targets to facilitate its potential application in the pharmaceutical market. However, it is noteworthy that overexpression of the *GGPPS* and *CPPS* genes also significantly boosted the accumulation of ferruginol and carnosic acid, two compounds with several interesting biological activities. Ferruginol and 1-oxo-ferruginol show antitumor activity against prostate cancer cells by inducing apoptosis (Bispo de Jesus et al., 2008), cytotoxicity against human pancreatic tumor cell lines (Fronza et al., 2011), and anti-inflammatory activity (Chen et al., 2011). Carnosic acid is a phenolic diterpene produced in a number of species belonging to the Lamiaceae family, including *Salvia* species, with anti-tumor, anti-diabetic, antibacterial, and neuroprotective properties (Birtić et al., 2015; Samarghandian et al., 2018). The results presented here also contribute to better understanding the metabolic constraints limiting the accumulation of these two pharmacologically interesting diterpenes synthesized in other plant species.

Metabolic engineering is successful only when modifications in the metabolic pathway do not interfere pleiotropically with the biosynthesis of other compounds and plant growth and development, a relevant aspect that is often under-evaluated in metabolic engineering strategies (Skraly et al., 2018). Overexpression of *GGPPS* and *CPPS* genes, individually or in combination, only slightly affected the final dry weight of transgenic *S. sclarea* HRs (**Figure S6**) and it was possible to screen HR lines with enhanced transcript levels of these two genes coupled to a profitable final yield of aethiopinone. The best performing lines in terms of aethiopinone and AD total yield were those overexpressing the *CPPS* gene individually, but we do not exclude the possibility of screening additional co-expressing lines with an unbiased ratio between precursor pools and enzymatic activities to achieve an additive/synergistic effect on aethiopinone accumulation.

## Conclusions

In conclusion, the results described here, along with our previously published findings, demonstrate that it is feasible to enhance the production of aethiopinone and other ADs in *S. sclarea* HRs by engineering different biosynthetic genes acting at controlling hubs of the terpenoid pathway. We have demonstrated that the overexpression of the *GGPPS* or *CPPS* gene is a highly efficient approach to enhance the total content of aethiopinone (8-fold and 10-fold increase in *GGPPS* and *CPPS* overexpressing HR lines, respectively). To the best of our knowledge, this is the first report of metabolic engineering based on the overexpression of the *CPPS* gene to boost the synthesis of ADs in *S. sclarea*, a strategy that can also be extended to engineering other class of diterpenes sharing CPP as a substrate (carnosic acid, ferruginol, and others) in different *Salvia* spp. It was also evident from our data that a higher availability of the GGPP pool, obtained either by inhibiting the gibberellin competitive branch route, by RNA*i*-mediated silencing, or by *GGPPS* overexpression is essential to drive this common precursor towards a higher accumulation of ADs. Cracking the biosynthetic route from CPP to aethiopinone by identifying the subsequent enzymatic steps will complement instrumentally the data presented here and our previous knowledge information on the contribution of other biosynthetic genes of the plastidial MEP-pathway, such as DXS and DXR, to the rationale design of a production platform to yield reliable amounts of bioactive abietane diterpenes in *S. sclarea* HRs.

## Data Availability Statement

The datasets generated for this study can be found in https://www.ncbi.nlm.nih.gov/nuccore/MK442922, https://www.ncbi.nlm.nih.gov/nuccore/MK442923, https://www.ncbi.nlm.nih.gov/nuccore/MK517475.

## Author Contributions

AL, MV, and MA conceived the study and designed the experiments. MA and MV performed the experiments. MA and MV constructed plasmids for hairy root transformation, and analyzed gene expression and hairy growth analysis. NT performed the HPLC–DAD quantitative analysis and analyzed metabolic data. MA, MV, and AL analyzed the data, wrote the manuscript, and prepared the figures. All authors contributed to the article and approved the submitted version.

## Conflict of Interest

The authors declare that the research was conducted in the absence of any commercial or financial relationships that could be construed as a potential conflict of interest.

## Supplementary Material

Figure S1 **(A)** PCR amplification of *entCPPS* using different couples of degenerate primers starting from *S. sclarea* hairy root cDNA pool. The white circle indicates the amplicon (expected size of 518 bp), that was purified, cloned into pCR2.1 vector, and sequenced. **(B)** Alignment of deduced amino acid sequence with the protein sequence of *Arabidopsis thaliana* (At4g02780). **(C)** 3′-RACE PCR experiments. On the basis of the partial sequence obtained, a specific primer was designed for extending the coding sequence. An amplicon of 1821 bp was purified, cloned into pCR2.1 vector, and sequenced (GeneBank Accession MK517475). **(D)** Multiple alignment of deduced amino acid sequences of *Salvia sclarea entCPPS*. Dark shading and gray shading indicate identical and similar amino acid residues, respectively. Conserved DIDD motifs, corresponding to the enzymatic synthase activity, reported in black and evidenced by a red box. Accession numbers: *Glycine max* (XP_003520571); *Populus trichocarpa* (XP_002302110); *Ricinus communis* (XP_002520733); *Arabidopsis thaliana* (At4g02780); **(E)** Schematic representation of the plasmid construct used for the RNAi-mediated silencing of *S. sclarea entCPPS* gene. The transcription is driven by the constitutive strong viral 35SCaMV promoter. *NPTII* (resistance to kanamycin); LB, left border; RB, right border; attb1 and attb2, homologous recombination sites. **(F)** Molecular characterization of *entCPPS* silenced hairy root lines. PCR amplification of genomic DNA using specific primers for *NPTII* and *RolB* genes. Genomic DNA was also used to amplify the *VirD2* gene to confirm the absence of contaminating bacteria. C^+^, *A. rhizogenes* plasmid carrying the RNA*i* construct.

Figure S2 CLUSTALW multiple protein sequence alignment of *Salvia sclarea* Ss*GGPPS* (MK442922) *Salvia miltiorrhiza*, SmGGPPS (ACJ66778.1), *Catharanthus roseus* CrGGPPS (AEI53622.1), *Arabidopsis thaliana*, AtGGPPS (AAM65107.1), *Corylus avellana*, CaGGPPS (ABW06960.1), and *Nicotiana tabacum*, NtGGPPS (ADD49734.1). Identical amino acid residues are indicated with a gray background. The SsGGPPS plastidial signal peptide is indicated with a box. The typical prenyl-transferase family domains (I–V) are indicated with a number and a gray background; the aspartate-rich box present in domain II (DDXXXXD) and in domain V (DDXXD) are indicated in white with a black background and underlined.

Figure S3 CLUSTALW multiple protein sequence alignment of *Salvia sclarea* SsCPPS (MK442923), *Salvia miltiorrhiza* (ABV57835.1), *Rosmarinus officinalis* (AHL67261.1), *Salvia fruticosa* (AJQ30184.1), *Helianthus annuus* (cbl42915.1*), Populus thrichocarpa* (XP_002302110.1), and *Solanum lycopersicum* (AEP82766.1*)*. The SsCPPS was also aligned with the *Salvia sclarea* Labd-13-en-8-ol diphosphate synthase (LPPS, AET21247.1), the enzyme leading to the synthesis of sclareol in the flowers. Identical amino acid residues are indicated with asterisk. The SsCPPS plastidial signal peptide is underlined in red. The aspartate-rich DIDD box is indicated by a red box.

Figure S4 Molecular characterization of *GGPPS, CPPS, and GGPPS/CPPS overexpressing* root lines. PCR amplification of genomic DNA using specific primers for *NPTII* and *RolB* genes. Genomic DNA was also used to amplify the *VirD2* gene to confirm the absence of contaminating bacteria. EV, empty vector transformed root line; C^+^, *A. rhizogenes* plasmid carrying the RNA*i* construct.

Figure S5 Chemical structures of principle abietane diterpenes synthetized in the roots of *Salvia sclarea* (upper panel). Comparison of the chromatograms of control hairy root line **(A)** with representative transformed hairy root lines *SsCPPS* **(B)**, *SsGGPPS* **(C)**, *SsCPPS/GGPPS* **(D)**, and *SsEntCPPS* **(E)**.

Figure S6 Biomass production expressed as dry weight of three independent hairy root lines overexpressing different levels of the biosynthetic genes are compared to EV (empty vector) hairy root line, during four 4 weeks of culture. Data represent mean values ± sd of three experimental replicates for each transgenic hairy root. **(A)** Chlorocholine-chloride (CCC) elicited roots; **(B)** *entCPPS* silenced hairy root lines; **(C)** *CPPS* overexpressing hairy root lines; **(D)** *GGPPS* overexpressing hairy root lines; **(E)** *CPPS/GGPPS* overexpressing hairy root lines. (*** P ≤ 0.001).
