# Supplementary figures and images for: Boosting the Synthesis of Pharmaceutically Active Abietane Diterpenes in S. sclarea Hairy Roots by Engineering the GGPPS and CPPS Genes
Source: Front Plant Sci. 2020 Jun 18;11:924. doi: 10.3389/fpls.2020.00924 (PMC7315395; doi:10.3389/fpls.2020.00924)

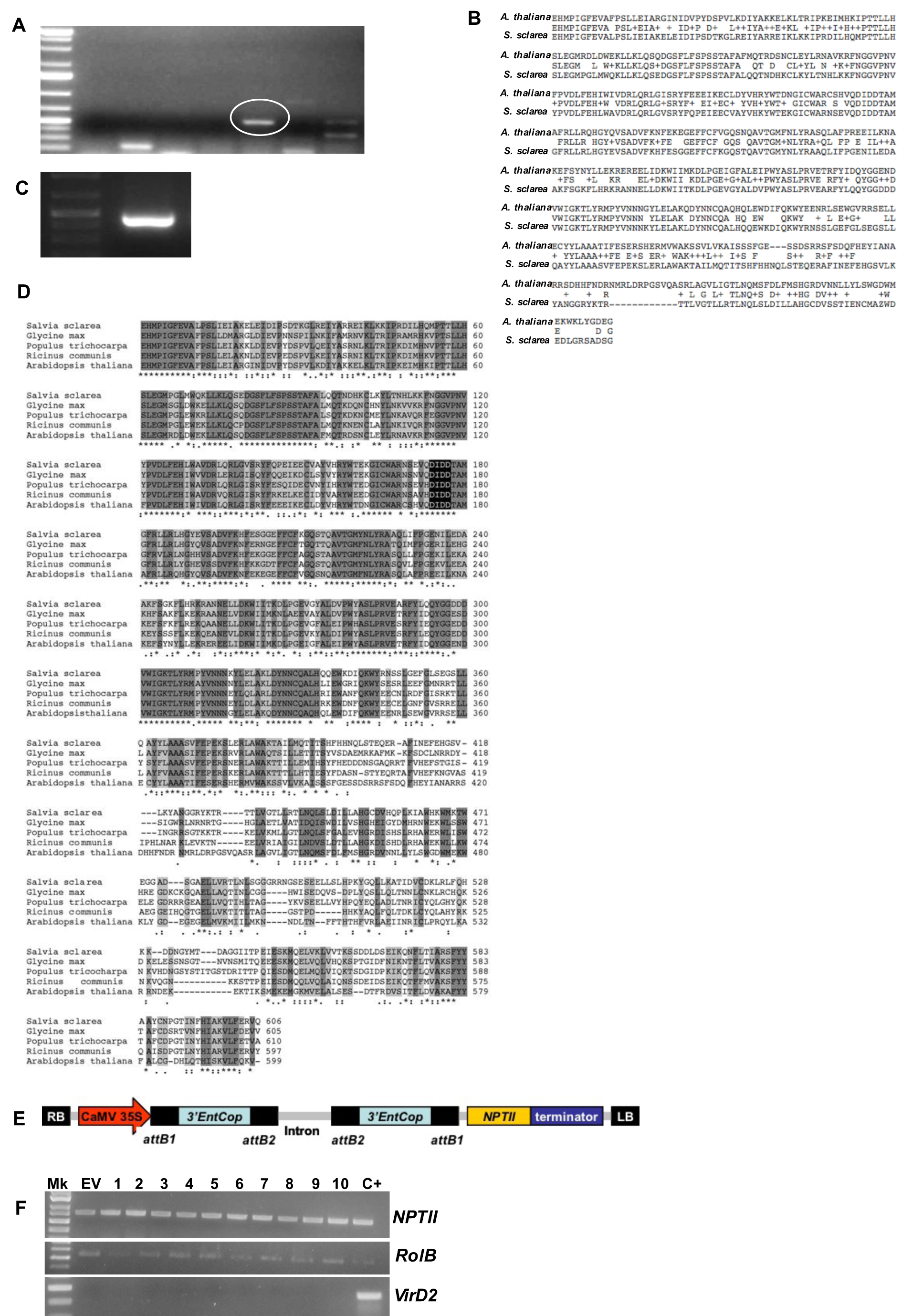

Supplement: Figure S1 — (A) PCR amplification of entCPPS using different couples of degenerate primers starting from S. sclarea hairy root cDNA pool. The white circle indicates the amplicon (expected size of 518 bp), that was purified, cloned into pCR2.1 vector, and sequenced. (B) Alignment of deduced amino acid sequence with the protein sequence of Arabidopsis thaliana (At4g02780). (C) 3′-RACE PCR experiments. On the basis of the partial sequence obtained, a specific primer was designed for extending the coding sequence. An amplicon of 1821 bp was purified, cloned into pCR2.1 vector, and sequenced (GeneBank Accession MK517475). (D) Multiple alignment of deduced amino acid sequences of Salvia sclarea entCPPS. Dark shading and gray shading indicate identical and similar amino acid residues, respectively. Conserved DIDD motifs, corresponding to the enzymatic synthase activity, reported in black and evidenced by a red box. Accession numbers: Glycine max (XP_003520571); Populus trichocarpa (XP_002302110); Ricinus communis (XP_002520733); Arabidopsis thaliana (At4g02780); (E) Schematic representation of the plasmid construct used for the RNAi-mediated silencing of S. sclarea entCPPS gene. The transcription is driven by the constitutive strong viral 35SCaMV promoter. NPTII (resistance to kanamycin); LB, left border; RB, right border; attb1 and attb2, homologous recombination sites. (F) Molecular characterization of entCPPS silenced hairy root lines. PCR amplification of genomic DNA using specific primers for NPTII and RolB genes. Genomic DNA was also used to amplify the VirD2 gene to confirm the absence of contaminating bacteria. C+, A. rhizogenes plasmid carrying the RNAi construct. [file Image_1.tif]

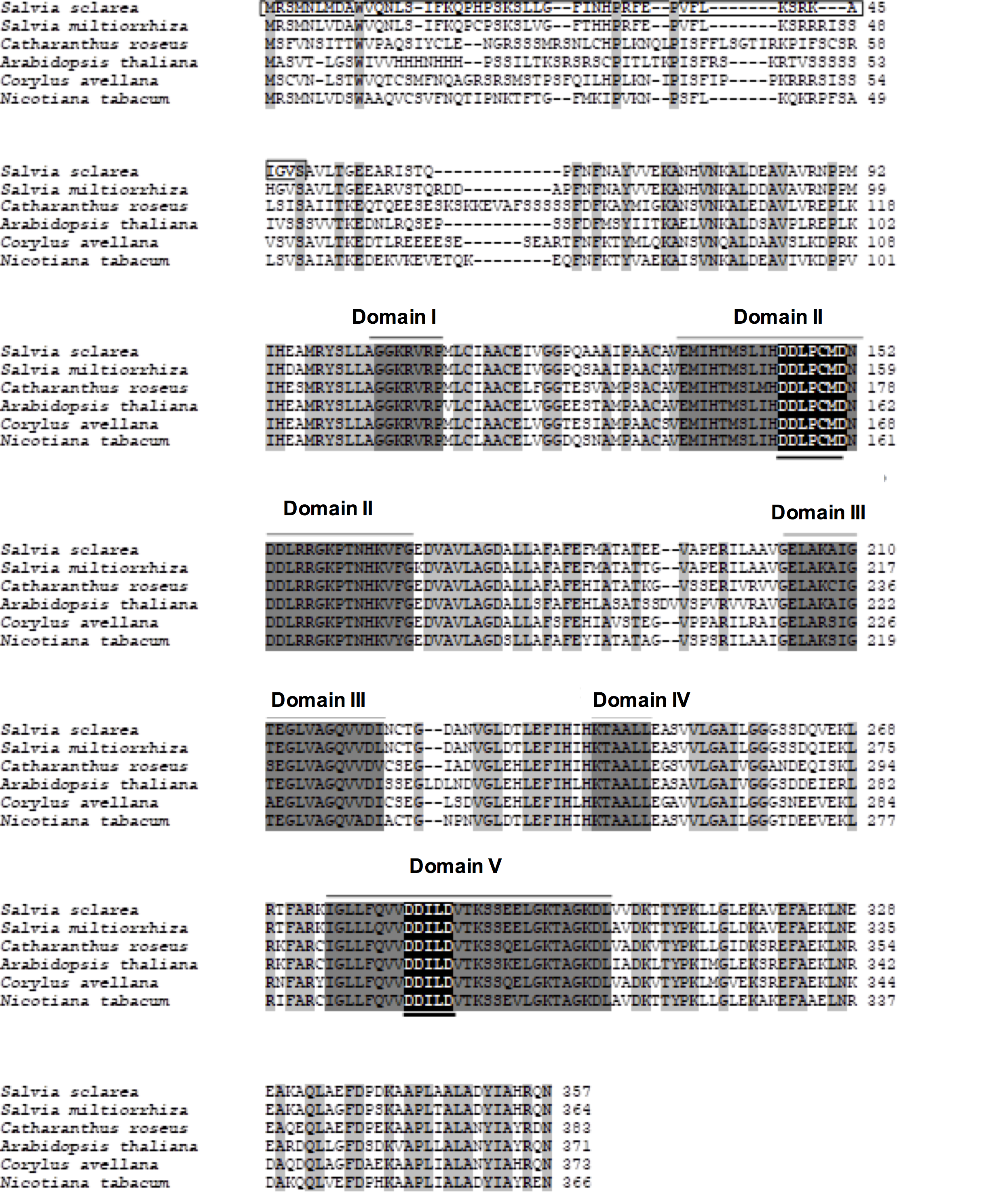

Supplement: Figure S2 — CLUSTALW multiple protein sequence alignment of Salvia sclarea SsGGPPS (MK442922) Salvia miltiorrhiza, SmGGPPS (ACJ66778.1), Catharanthus roseus CrGGPPS (AEI53622.1), Arabidopsis thaliana, AtGGPPS (AAM65107.1), Corylus avellana, CaGGPPS (ABW06960.1), and Nicotiana tabacum, NtGGPPS (ADD49734.1). Identical amino acid residues are indicated with a gray background. The SsGGPPS plastidial signal peptide is indicated with a box. The typical prenyl-transferase family domains (I–V) are indicated with a number and a gray background; the aspartate-rich box present in domain II (DDXXXXD) and in domain V (DDXXD) are indicated in white with a black background and underlined. [file Image_2.tiff]

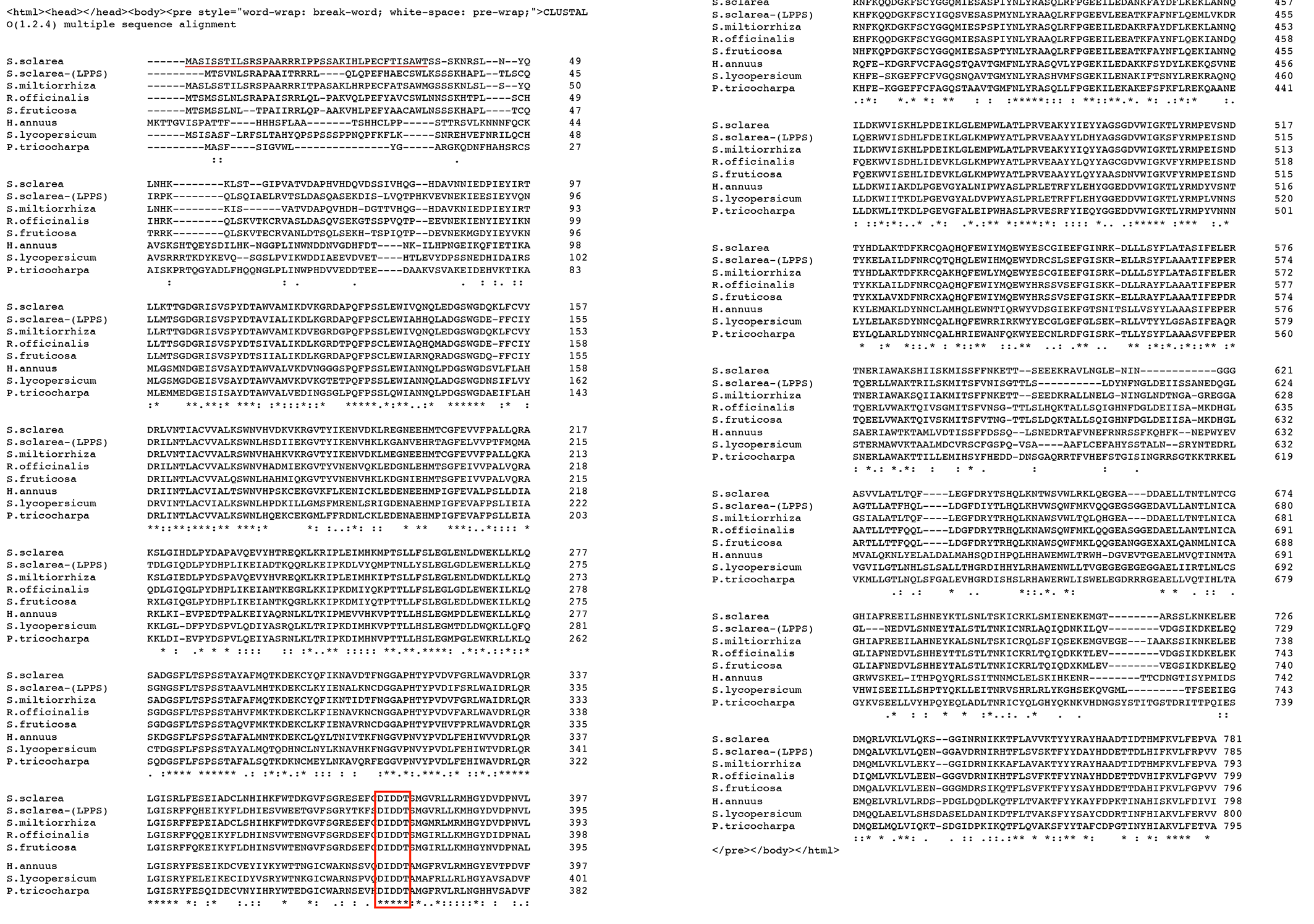

Supplement: Figure S3 — CLUSTALW multiple protein sequence alignment of Salvia sclarea SsCPPS (MK442923), Salvia miltiorrhiza (ABV57835.1), Rosmarinus officinalis (AHL67261.1), Salvia fruticosa (AJQ30184.1), Helianthus annuus (cbl42915.1), Populus thrichocarpa (XP_002302110.1), and Solanum lycopersicum (AEP82766.1). The SsCPPS was also aligned with the Salvia sclarea Labd-13-en-8-ol diphosphate synthase (LPPS, AET21247.1), the enzyme leading to the synthesis of sclareol in the flowers. Identical amino acid residues are indicated with asterisk. The SsCPPS plastidial signal peptide is underlined in red. The aspartate-rich DIDD box is indicated by a red box. [file Image_3.tif]

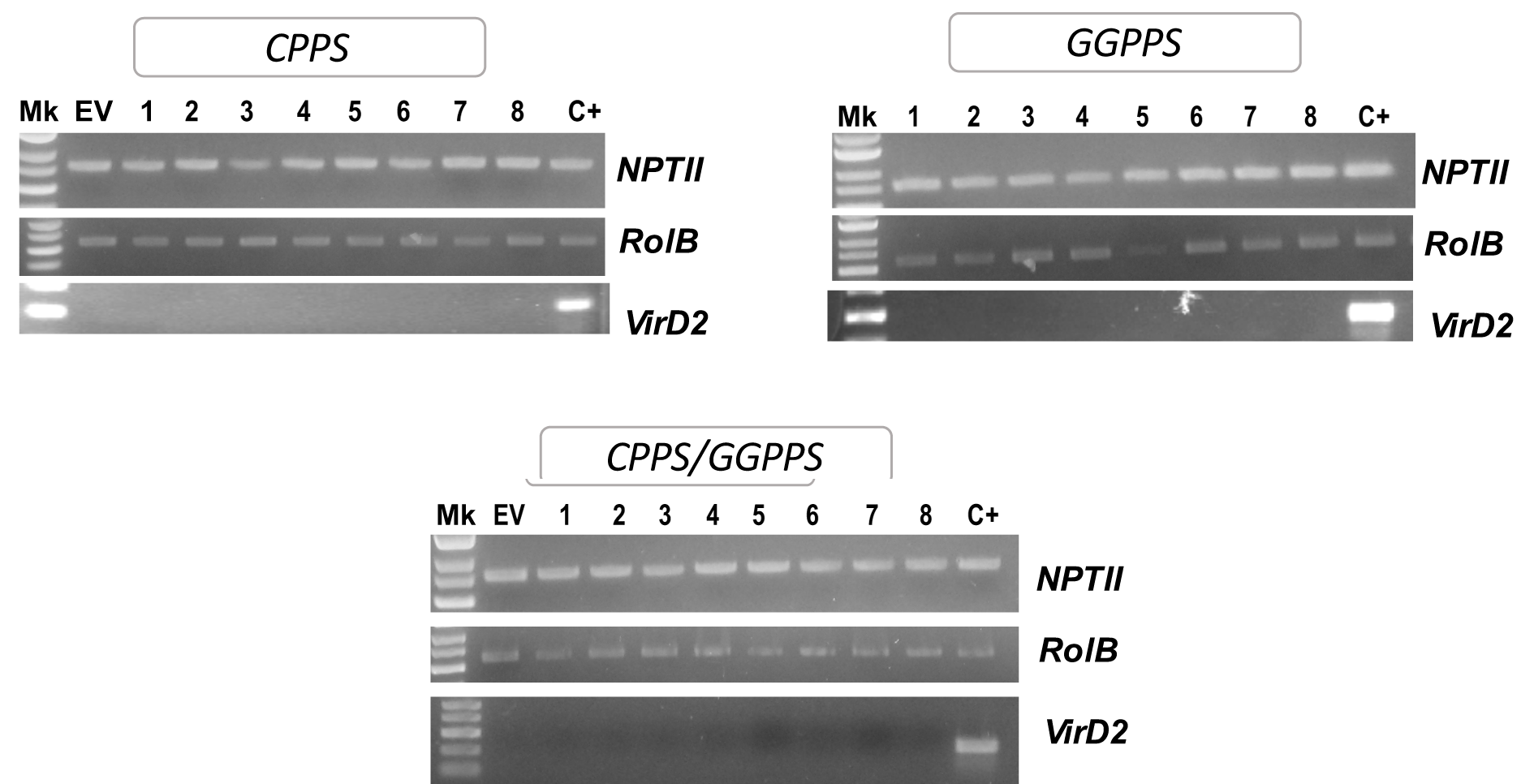

Supplement: Figure S4 — Molecular characterization of GGPPS, CPPS, and GGPPS/CPPS overexpressing root lines. PCR amplification of genomic DNA using specific primers for NPTII and RolB genes. Genomic DNA was also used to amplify the VirD2 gene to confirm the absence of contaminating bacteria. EV, empty vector transformed root line; C+, A. rhizogenes plasmid carrying the RNAi construct. [file Image_4.tif]

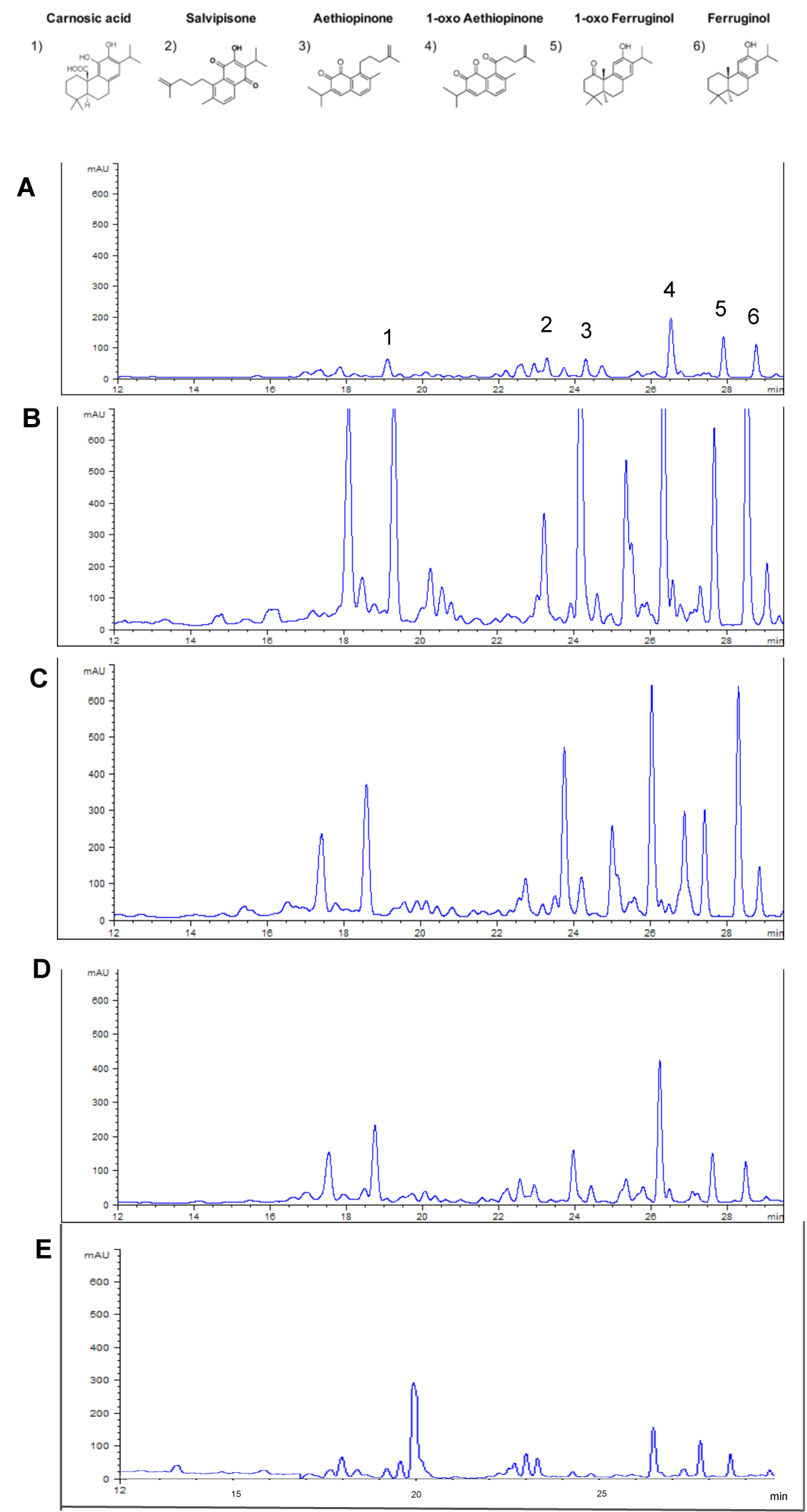

Supplement: Figure S5 — Chemical structures of principle abietane diterpenes synthetized in the roots of Salvia sclarea (upper panel). Comparison of the chromatograms of control hairy root line (A) with representative transformed hairy root lines SsCPPS (B), SsGGPPS (C), SsCPPS/GGPPS (D), and SsEntCPPS (E). [file Image_5.tif]

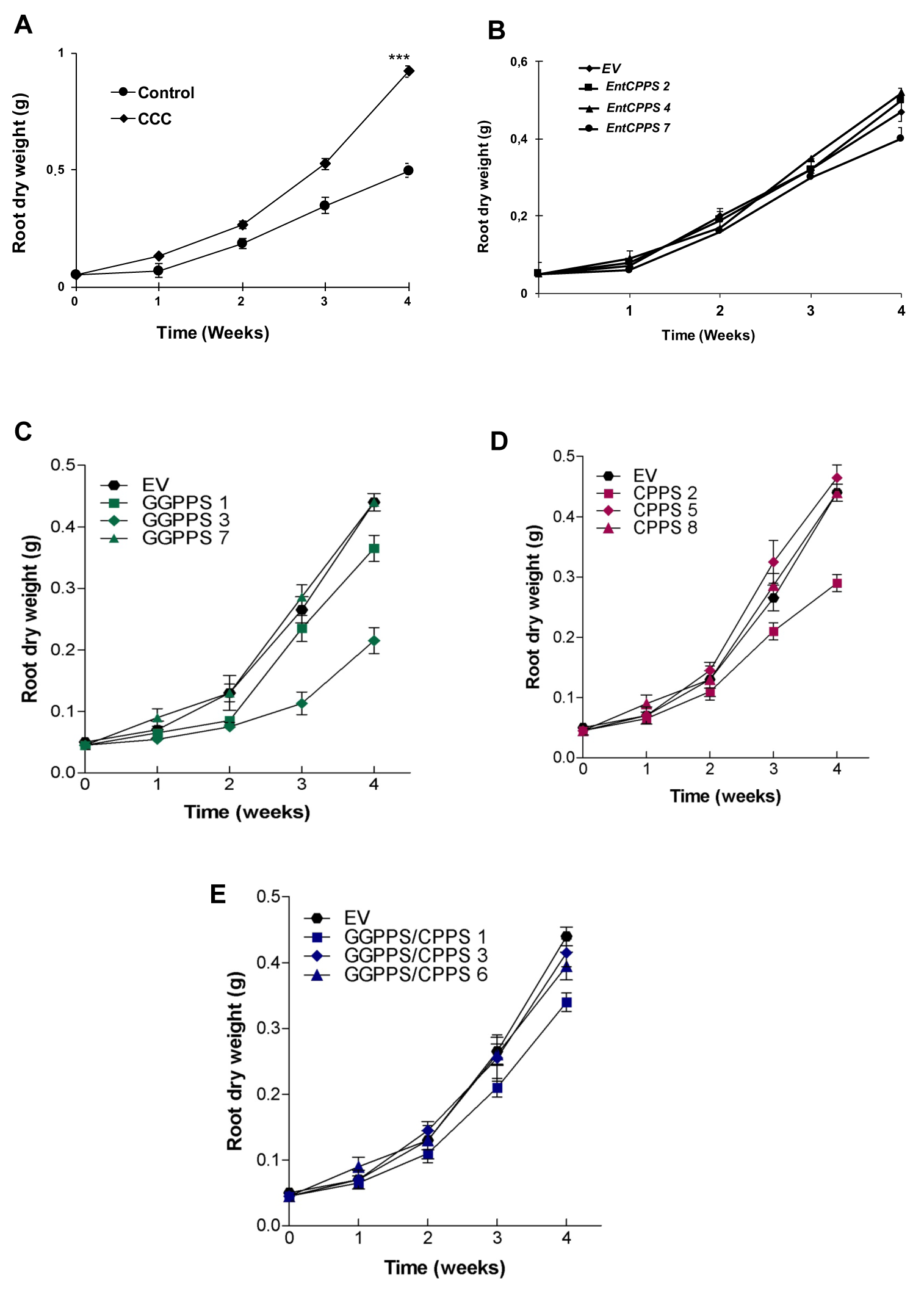

Supplement: Figure S6 — Biomass production expressed as dry weight of three independent hairy root lines overexpressing different levels of the biosynthetic genes are compared to EV (empty vector) hairy root line, during four 4 weeks of culture. Data represent mean values ± sd of three experimental replicates for each transgenic hairy root. (A) Chlorocholine-chloride (CCC) elicited roots; (B) entCPPS silenced hairy root lines; (C) CPPS overexpressing hairy root lines; (D) GGPPS overexpressing hairy root lines; (E) CPPS/GGPPS overexpressing hairy root lines. (*** P ≤ 0.001). [file Image_6.tif]
