# Supplementary material for: Boosting the Synthesis of Pharmaceutically Active Abietane Diterpenes in S. sclarea Hairy Roots by Engineering the GGPPS and CPPS Genes
Source: Front Plant Sci. 2020 Jun 18;11:924. doi: 10.3389/fpls.2020.00924 (PMC7315395; doi:10.3389/fpls.2020.00924)
Supplement: Supplementary file 7 [file Table_1.doc]

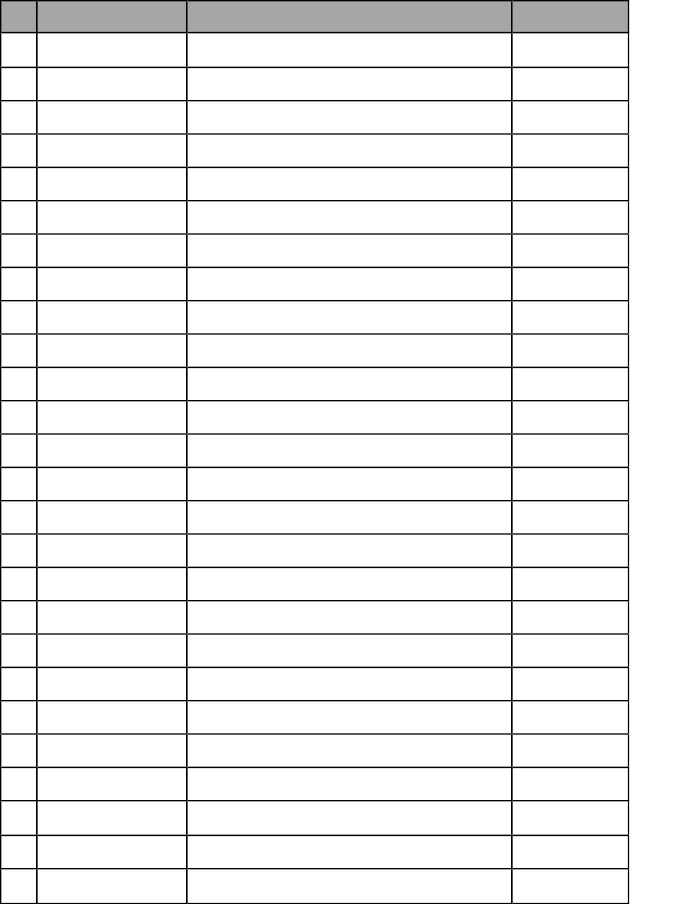
Name

1

2

3

5

5

6

7

8

9

10

11

12

13

14

15

16

17

18

25

26

26

27

28

29

30

31

RT-SsGGPPS-F

RT-SsGGPPS-R

RT-SsCPPS-F

RT-SsCPPS-F

RT-SsActin-F

RT-SsActin-R

Ss-18S-F

Ss-18S-R

SmGGPPS284-F

SmGGPPS449-F

SmGGPPS 693-R

SmGGPPS 898-R

SmCPPS346-F

SmCPPS 1316-R

SmCPPS 482-F

SmCPPS1255-R

SmCPPS 752-F

Sequences 5ʹ- 3ʹ

GTGGTGGACATCAACTGCAC

AAAATGGCCCCCAAAACTAC

GCGAAGACCGATTTCAAGAG

CAGTCGCCAGGAAATAGGAA

GGTGCCCTGAGGTCCTGTT

GAGCCACCACTGAGGACAAT

GCGTCGGACGTCTATCAAAT

TTGCGTTCAAAGACTCGATG

GGAATCCACCGATGATCCAC

TCCACGACGATCTACCCTGT

GCAGTTCAGGTCCACCACTT

CCGAAGACTTGGTCACATCC

AGGACGTTGTTGAGGACGAC

GAAGGCGACTCGATCATCTG

ATCAACTCGAGGATGGATCG

TCCTCAGCACATTTGGATCA

CGGTGCAGGAGGTTTATCAT

Use

qRT-PCR

qRT-PCR

qRT-PCR

qRT-PCR

qRT-PCR

qRT-PCR

qRT-PCR

qRT-PCR

RT-PCR

RT-PCR

RT-PCR

RT-PCR

RT-PCR

RT-PCR

RT-PCR

RT-PCR

RT-PCR

RT-PCR

3’ Race

5’ Race

3’ Race

3’ Race

5’ Race

RT-PCR

RT-PCR

3’ Race

Copalyl S.m 1255 Rev TCCTCAGCACATTTGGATCA

SsGGPPS 450-F

SsGGPPS 486-R

SsCPPS-Sp1-F

SsCPPS-Sp2-F

SsCPPS133-R

degEntCopFw

degEntCop319Rev

3RaceEntCop

CCACGACGATCTACCCTGT

CCCAAAACTACAGAAGCCTCTAGC

5’-CTTCTCGCCTTTTTGAGTCG-3’

5’-TTGATCCAAATGTGCTGAGG-3’

5’-CGTCACTCCTCTCTTAACCTTGTC-3’

GARCAYATGCCNATHGGNTTYGARGT

TCNACNGGRTANACRTTNGGNACNCC

GCAGAGACTGGGAGTTTCGCGTTATT
